# Wound of the Brain—Recovery

**Published:** 1849-10

**Authors:** William Kenney

**Affiliations:** Millersburg, Kentucky


ART. V.—*Wound of the Brain—recovery.* By WILLIAM KENNEY, M. D., of Millersburg, Kentucky.

Isaac Dubler, aged about seventeen, received, in an affray on the night of the 25th July, a stab with a common pocket knife, blade two inches and a half in length and three quarters of an inch in width, tapering abruptly on back and edge to a point. The wound was in the left temporal region. The knife was driven with such force as to penetrate the brain the full length of the blade, at a point midway and about three quarters of an inch above a line drawn from the external angle of the eye to the *meatus auditorius internus.* The handle of the knife as it stood looked slightly forwards and upwards, and was so firmly fixed between the divided bone that it was with great difficulty that the knife was removed. During its presence in the brain, and after its removal, the patient complained of great pain in the left eye and over the frontal region of that side. Its removal was followed by hemorrhage, to the amount of ten or twelve ounces, vomiting and stupor. The patient was taken from the street, his wound washed, and without farther dressings being applied, was placed in bed, with the head raised, and ordered the free use of cold applications to scalp; sinapisms to the extremities; xx. grs. calomel; rest and quiet.

26th. Patient somewhat restless during the night. Pain, drowsiness and vomiting persisted. Pulse 84, and slightly increased in force; face flushed; extremities warm; some intolerance of light, and slight conjunctival discoloration; urine scanty; tongue coated; parts around the wound swollen. No action on bowels.

Ordered an enema. The room to be darkened.—Light diet. A repetition of the calomel at night; continue cold application to the shaven scalp; sinapisms to the extremities.

27th. Patient restless; pulse 92; tongue red at edges and tip; some heat, and complains of pain of the head; intolerance of light and noise; continues drowsy with a sense of numbness of the entire left half of the body, but less disposition to vomit. Slight abdominal tenderness.

Ordered an enema. Blue mass and pulv. Dov. ää grs. v. at night; fomentations to the abdomen; iced mucilaginous drinks ; sinapisms, cold applications, etc., continued.

28th. Pulse 84; rested well; less pain of head and intolerance of light; tongue less red; abdominal tenderness diminished; drowsiness subsiding; wound less swollen, healthy and closing; numbness confined to the left half of the face; talks cheerfully; keeps awake, and wants to sit up; appetite increasing.

Prescriptions continued.

29th. Pulse 74; slept well during the night; skin moist; sits up; free from pain or uneasiness; feels weak; appetite good; wound closed, and patient says he is well.

31st. Patient still improving; walks about the room; feels vertigo occasionally, and still has the numb sensation of the left half of the face.

August 5th. Walking about town. Bowels regular; appetite good.

14. Discharged.

